# Genomic characterization of the bacterial phylum *Candidatus* Effluviviacota, a cosmopolitan member of the global seep microbiome

**DOI:** 10.1128/mbio.00992-24

**Published:** 2024-07-09

**Authors:** Lei Su, Ian P. G. Marshall, Andreas P. Teske, Huiqiang Yao, Jiangtao Li

**Affiliations:** 1 State Key Laboratory of Marine Geology, Tongji University, Shanghai, China; 2 Department of Biology, Center for Electromicrobiology (CEM), Section for Microbiology, Aarhus University, Aarhus, Denmark; 3 Department of Earth, Marine and Environmental Sciences, University of North Carolina at Chapel Hill, Chapel Hill, North Carolina, USA; 4 MLR Key Laboratory of Marine Mineral Resources, Guangzhou Marine Geological Survey, Guangzhou, China; Institut Pasteur, Paris, France

**Keywords:** cold seep, global distribution, bacteria, *Candidatus* Effluviviacota

## Abstract

The microbial communities of marine seep sediments contain unexplored physiological and phylogenetic diversity. Here, we examined 30 bacterial metagenome-assembled genomes (MAGs) from cold seeps in the South China Sea, the Indian Ocean, the Scotian Basin, and the Gulf of Mexico, as well as from deep-sea hydrothermal sediments in the Guaymas Basin, Gulf of California. Phylogenetic analyses of these MAGs indicate that they form a distinct phylum-level bacterial lineage, which we propose as a new phylum, *Candidatus* Effluviviacota, in reference to its preferential occurrence at diverse seep areas. Based on tightly clustered high-quality MAGs, we propose two new genus-level candidatus taxa, *Candidatus* Effluvivivax and *Candidatus* Effluvibates. Genomic content analyses indicate that *Candidatus* Effluviviacota are chemoheterotrophs that harbor the Embden–Meyerhof–Parnas glycolysis pathway. They gain energy by fermenting organic substrates. Additionally, they display potential capabilities for the degradation of cellulose, hemicellulose, starch, xylan, and various peptides. Extracellular anaerobic respiration appears to rely on metals as electron acceptors, with electron transfer primarily mediated by multiheme cytochromes and by a flavin-based extracellular electron transfer (EET) mechanism that involves NADH-quinone oxidoreductase-demethylmenaquinone-synthesizing enzymes, uncharacterized membrane proteins, and flavin-binding proteins, also known as the NUO-DMK-EET-FMN complex. The heterogeneity within the *Ca*. Effluviviacota phylum suggests varying roles in energy metabolism among different genera. While NUO-DMK-EET-FMN electron transfer has been reported predominantly in Gram-positive bacteria, it is now identified in *Ca*. Effluviviacota as well. We detected the presence of genes associated with bacterial microcompartments in *Ca*. Effluviviacota, which can promote specific metabolic processes and protect the cytosol from toxic intermediates.

**IMPORTANCE:**

The newly discovered bacterial phylum *Candidatus* Effluviviacota is widespread across diverse seepage ecosystems, marine environments, and freshwater environments, with a notable preference for cold seeps. While maintaining an average abundance of approximately 1% in the global gene catalog of cold seep habitats, it has not hitherto been characterized. The metabolic versatility of *Ca*. Effluviviacota in anaerobic carbon, hydrogen, and metal cycling aligns with its prevalence in anoxic niches, with a preference for cold seep environments. Variations in metabolic potential between *Ca*. Effluvivivax and *Ca*. Effluvibates may contribute to shaping their respective habitat distributions.

## INTRODUCTION

Direct sequencing of environmental DNA has shown that most microbial lineages have not been isolated in pure culture, and their physiologies have only been inferred from genomics and environmental characteristics (1). As-yet-uncultivated taxa represent approximately 85% of prokaryotic phylogenetic diversity (2), while named prokaryotes account for less than 0.2% of total species (3). Advances in DNA sequencing and computational methods have revealed many new lineages on the tree of life and have greatly expanded our understanding of uncultured microbes (4–6). Over the past decade, improvements in sequencing techniques and bioinformatics tools have revolutionized the way uncultured microorganisms can be classified. Binning single-population genomes from metagenomes and amplifying genome segments from single cells now dominate the identification of candidate taxa, transforming it from a data-poor into a data-rich field (7).

Cold seeps represent deep-sea havens that foster extensive microbial biodiversity (8). Previous studies based on amplicon and metagenomic sequencing have elucidated the phylogenetic diversity of archaeal and bacterial lineages in marine cold seep ecosystems (9, 10). These studies have also explored variations in the prokaryotic community structure and metabolic function across different regions and depth profiles within specific seepage areas (8, 11–14). A comprehensive study of globally distributed cold seep microbiomes has revealed the coexistence of both global and site-specific bacterial and archaeal groups in sediments, indicating local diversification of widely dispersed marine cold seep microbiomes (11). At the local scale, seep fluids rise vertically through the sediment, forming steep geochemical and microbial gradients; these gradients dissipate horizontally with increasing distance from the active center (1, 8). Changes in the composition of prokaryotic communities along these geochemical gradients have been attributed to changes in the availability of electron acceptors (9, 15) and the distribution of hydrocarbons, including methane (16, 17).

Nonetheless, gaps in the identification of active microbial taxa, their metabolism, and biogeochemical relevance limit our understanding of the seep biosphere. Consistently, genome-based diversity surveys have unveiled the presence of numerous unexplored taxa (12, 14, 18), emphasizing that a significant proportion of microorganisms in seep environments remains uncharacterized (6, 12, 19). In particular, some bacterial and archaeal lineages, such as the cosmopolitan phylum-level bacterial lineage VGIX01 (classified by GTDB), are widespread and account for approximately 1% of the cold seep microbiomes in the global cold seep gene catalog (19). However, little is known about its distribution, function, and the reasons for its prevalence in global cold seep ecosystems. Here, we report on the genomic characteristics, including inferred metabolic capacities, physiological preferences, structural features, and ecological distribution of a cosmopolitan preferentially found in marine hydrocarbon seeps.

## MATERIALS AND METHODS

### Sediment collection, DNA extraction, and sequencing

Sediments of this study were collected from the Haima cold seep (HM2 and HM3) in the South China Sea, the Makran cold seep in the Indian Ocean, and hydrothermal sediments of the Guaymas Basin in the Gulf of California (Tables S1 and S2). Sampling information and measurements of various geochemical parameters of sediments from the Haima and Guaymas Basin were specified in our recent reports (20, 21). The Makran cold seep sediment samples were collected during the China–Pakistan joint marine scientific cruise in 2018–2019, and the measurement of methane and sulfate concentrations followed the methodology outline by Liu et al. (20). The geochemical profiles of these three sediment sites are compiled together in Fig. S1.

Total nucleic acids were extracted from subsamples of the vertically profiled sediments with the Fast DNA Spin Kit for Soil (MP Biomedicals, Santa Ana, CA) according to the manufacturer’s recommendations. Nucleic acid extracts were stored at −80°C until further processing. Sequencing libraries were generated using the NEB Next Ultra DNA Library Prep Kit for Illumina (New England Biolabs, MA, USA) following the manufacturer’s protocol, and index codes were added simultaneously. Library quality was assessed using the Qubit 3.0 Fluorometer (Life Technologies, Grand Island, NY) and Agilent TapeStation 4200 system (Agilent, Santa Clara, CA). Finally, each library was sequenced on the Illumina NovaSeq platform (Illumina, USA) using the PE150 strategy at Personal Biotechnology Co., Ltd. (Shanghai, China) and Guangdong Magigene Biotechnology Co., Ltd. (Guangzhou, China).

### Metagenomic processing, assembly, and binning

We trimmed adapters and poor-quality sequences from the raw sequencing data using the default parameters of Cutadapt (v.4.1; https://github.com/marcelm/cutadapt) (22). After removing adapter sequences and poor-quality sequences (lengths less than 20 bp or average quality values less than Q20), all of the QC-filtered data were quality-checked with FastQC (v0.12.1; https://github.com/s-andrews/FastQC). The QC-filtered forward and reverse metagenomic sequencing libraries contained a range of 151,680,435–240,595,851 reads that were ~150 bp long, and approximately 45.50–72.18 Gbp of clean reads were obtained for each sample (Table S2).

The sequences after quality control were assembled separately using Megahit (v.1.2.9; https://github.com/voutcn/megahit) (23) with the following parameters: --min-count 2 k-min 41 --kmin-1pass --k-max 141 --k-step 10. Contigs longer than 1,000 bp were binned into genomes based on their nucleotide frequencies and coverage across samples. Assemblies were binned using MetaBAT2 (v.2.12.1), Maxbin2 (v.2.2.4), and CONCOCT (v.1.1.0) with default parameter settings in MetaWRAP (v1.2.1, https://github.com/bxlab/metaWRAP) (24). The resulting genome bins were further refined using the binning_refinement module in MetaWRAP to obtain high-quality metagenome-assembled genomes (MAGs).

Completeness, contamination, and the genome size of MAGs were estimated using CheckM2 (v.0.1.3; https://github.com/chklovski/CheckM2) (25). MAGs with completeness greater than 50% and contamination less than 10% were selected for further analysis, resulting in a total of 1,972 MAGs. The true genome size was estimated by dividing the size of the MAG by its estimated completeness and redundancy (indicating potential contamination or duplication) (26). Here, we focus on 14 specific MAGs that were identified as VGIX01 by the GTDB-TK reference database (version r207) (27). In addition, we also present the relative abundance of VGIX01 MAGs in the Haima cold seep sediments (Fig. S2).

### Collection of VGIX01 metagenomes

We collected available VGIX01 MAGs from the National Center for Biotechnology Information (NCBI) BioProject. All these data come from six globally distributed seep sites (including oil and gas seeps, as well as methane seeps), one hydrothermal vent field, one hypersaline water, and one freshwater site (Table S1). These sites include seeps in the Gulf of Mexico (GoM), the Scotian Basin (SB), in Site F (SF) and Jiaolong (JL) cold seeps in the South China Sea, Shark Bay in Australia, and Lake Tanganyika in Tanzania (Table S1). We also collected data from other studies of the Haima cold seep (HMR) (Table S1).

### Phylogenomic analyses

The amino acid identity (AAI) of all the VGIX01 MAGs and their references was estimated using CompareM (v0.1.2) aai_wf command (https://github.com/dparks1134/CompareM). The average nucleotide identity (ANI) between all genomes was calculated using fastANI (version 1.33; https://github.com/ ParBLiSS/ FastANI).

The MAG phylogeny was constructed using IQ-TREE with the following steps: First, the bacterial genome assemblies were aligned to the marker gene sequences of 120 bacteria in the GTDB-TK reference database (version r207) (27); each of the 120 marker protein sequences from the reference genomes and the MAGs was aligned using the MAFFT (28) algorithm version 7.525 with the parameters --ep 0 --genafpair --maxiterate 1,000 and filtered with trimal (v1.4. rev15) with parameter -automated1 (29). Then, all 120 marker genes were concatenated into a single alignment, and phylogenetic trees were constructed using both IQ-TREE (v.2.1.4; https://github.com/iqtree/iqtree2) (30) with the parameters -m MFP -b 100 -T 10 and the best-fit evolutional model (LG+F+R10). Finally, the resulting tree was visualized and edited using iTOL (https://itol.embl.de/).

16S rRNA gene sequences were extracted from all VGIX01 MAGs of the genomic tree using Barrnap (v0.9; https://github.com/tseemann/barrnap) (31) with default settings. All 16S rRNA gene sequences from retrieved genomes were aligned using ClustalW (v2.1). A maximum-likelihood phylogenetic tree of VGIX01 and its references was inferred using IQ-TREE with the best-fit evolutionary model and 1,000 bootstrap replicates. Only full-length 16S rRNA gene sequences were considered for subsequent analysis. 16S rRNA sequences were aligned with ClustalW. The numbers of divergent and identical nucleotide positions of 16S rRNA genes were determined. For divergent sequences, the percentage of nucleotide difference was calculated using MEGAX11 (v11.0.10; 32) with the Jukes–Cantor model (33).

We then submitted the complete 16S rRNA gene sequences of assembled genomes to the Microbe Atlas Project sequence (MAPseq; https://microbeatlas.org/) (34) to investigate the geographic distribution of this phylum. In addition, MAPseq provides fast and accurate sequence read mapping against hierarchically clustered and annotated reference sequences. 16S rRNA gene sequences from the MAGs matched reference sequences in MAPseq with >94% identity (35). We analyzed the MAPseq mapping results to visualize the global distribution of VGIX01 and similar sequences at Level 90 (order level).

### Metabolic predictions

Gene predictions for individual genomes were performed using Prokka (v1.14.5; https://github.com/tseemann/prokka) (36). Predicted genes from individual genomes were further characterized using a combination of several databases: KofamKOALA (v1.3.0), HydDB, and TIGRfam. For KofamKOALA, only hits above the predefined threshold for individual KOs were selected. Hydrogenases were annotated based on a web-based hydrogenase classifier, HydDB (http://services.birc.au.dk/hyddb/).

The METABOLIC (METabolic And BiogeOchemistry anaLyses In miCrobes) tool (37) was employed for annotating microbial genomes. To annotate carbohydrate-active enzymes (CAZymes), proteins were annotated using dbCAN2 with default settings. In addition, a non-redundant library of protein sequences encompassing all peptidase/inhibitor units from the peptidase database MEROPS was used as the reference database for the search against putative peptidases and inhibitors using the DIAMOND program (38) with an *E*-value <1*e*−5.

Outer-membrane cytochromes were predicted by scanning proteins with Interproscan version 5.60-92.0 (39). All multiheme cytochromes identified by Interproscan (accession IPR036280, Multiheme cytochrome superfamily) were then searched for signal peptides using SignalP 6.0 (https://services.healthtech.dtu.dk/services/SignalP-3.0/) (40).

## RESULTS AND DISCUSSION

### Genomic reconstruction and collection of VGIX01

This study resulted in the reconstruction of 14 draft MAGs with >50% completeness and <10% contamination that were affiliated with the VGIX01 group, including 10 MAGs (all MAGs beginning with 70 and 72) from Haima cold seep sediments (South China Sea), 3 MAGs (G352_bin.14, G355_bin.45, and G3515_bin.52) from Makran cold seep sediments (Northeastern Arabian Sea off Pakistan, Indian Ocean), and 1 MAG (GB53_bin.51) from Guaymas Basin hydrothermal sediments (Gulf of California) (Table S2). Significantly, all these MAGs are found in anoxic, methane-rich cold seep sediments and warm hydrothermal sediments, with the highest relative abundance of 5.1% in the Haima cold seep (Fig. S2).

Sixteen additional MAGs belonging to VGIX01 in the NCBI BioProject were downloaded for subsequent analysis in this study (Tables S1 and S2), two of them from Shark Bay hypersaline water (Hamelin Pool, Australia), one from Lake Tanganyika freshwater (Mahale, Tanzania), six from Haima cold seep sediments, two from Jiaolong cold seep sediments (South China Sea), two from Scotian Basin cold seeps, two from Site F cold seep sediments (South China Sea), and one from Gulf of Mexico sediment.

### General genomic features and phylogeny

The completeness of the resulting 30 MAGs was generally greater than 50%, and the converted genome size ranged from 2.14 to 5.10 Mbp, with the majority falling within the range of 2–4 Mb. Notably, only JL_Co_1 and 7246_bin.4 were larger than 4 Mb. The GC content of the genomes ranged from 58.82% to 70.35% (Fig. S3; Table S3).

**Fig 1:**
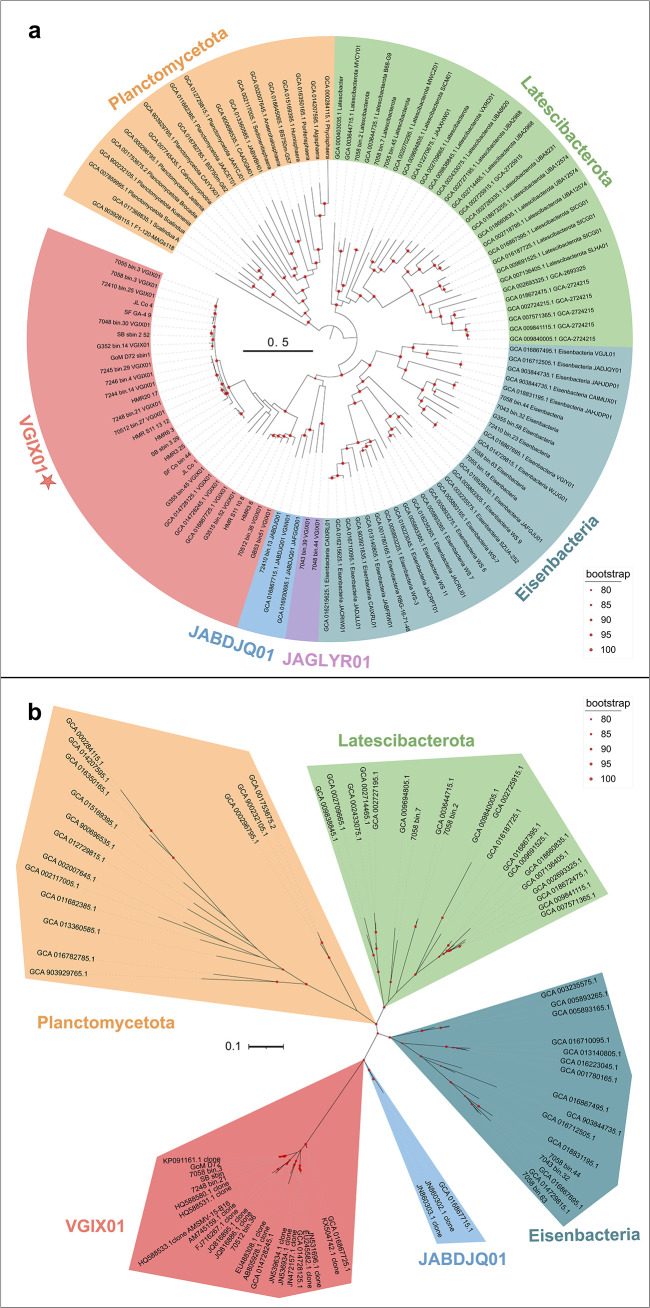
Phylogenetic differentiation of VGIX01 and related lineages. Maximum-likelihood phylogenies of VGIX01 and related lineages based on assembled metagenomes (a) and 16S rRNA gene sequences (b). The genome and 16S rRNA gene trees were inferred using IQ-TREE with the best-fit evolutionary models and 1,000 bootstrap replicates. Different color backgrounds represent different phyla. The red dots have been placed on the nodes to represent only those nodes with ≥80% support.

Phylogenetic analysis of the 30 MAGs within the VGIX01 cluster, based on a concatenated alignment of 120 conserved proteins (see Materials and Methods), placed into a deeply branched, strongly supported (100% bootstrap) bacterial lineage that is distinct from Eisenbacteria, Latescibacterota, the currently uncultured JABDJQ01 and JAGLYR01 lineages, and other known bacterial phyla (Fig. 1a; Fig. S4; Table S3). Interestingly, bootstrap analysis of these lineages supported a shared root for Latescibacteria, Eisenbacteria, JABDJQ01, JAGLYR01, and VGIX01 (Fig. S4). A shared root for these MAG-defined lineages suggests that they constitute a superphylum (41), but its precise delimitation requires further investigation beyond the scope of this study.

Phylogenetic analysis of the 16S rRNA gene sequences obtained from our MAGs indicated that the VGIX01 lineage is separated within the phylum Latescibacterota or other described bacterial phyla (Fig. S5 and S6). The average 16S rRNA gene divergence between VGIX01 and JABDJQ01 is 18.04%, between VGIX01 and Latescibacterota is 23.56%, and between VGIX01 and Eisenbacteria is 20.35% (Fig. S6; Table S13). These 16S rRNA gene divergences support the definition of these lineages as distinct phyla, given the suggested range of 75%–83% 16S rRNA gene sequence identity (25%–17% divergence) to separate phylum-level lineages (7). While bootstrap values strongly support VGIX01 as a distinct lineage (100%), they also suggest the possibility of a superphylum, indicated by 100% bootstrap support for the combined Latescibacterota, Eisenbacteria, JABDJQ01, and VGIX01 lineages (Fig. S5).

Overall, the results of the genome and 16S rRNA gene phylogenies indicate that these MAGs cannot be included within any previously described phylum but represent a separate, previously undescribed phylum-level lineage for which we propose the phylum name “*Candidatus* Effluviviacota” based on the International Code of Nomenclature of Prokaryotes.

**Fig 2:**
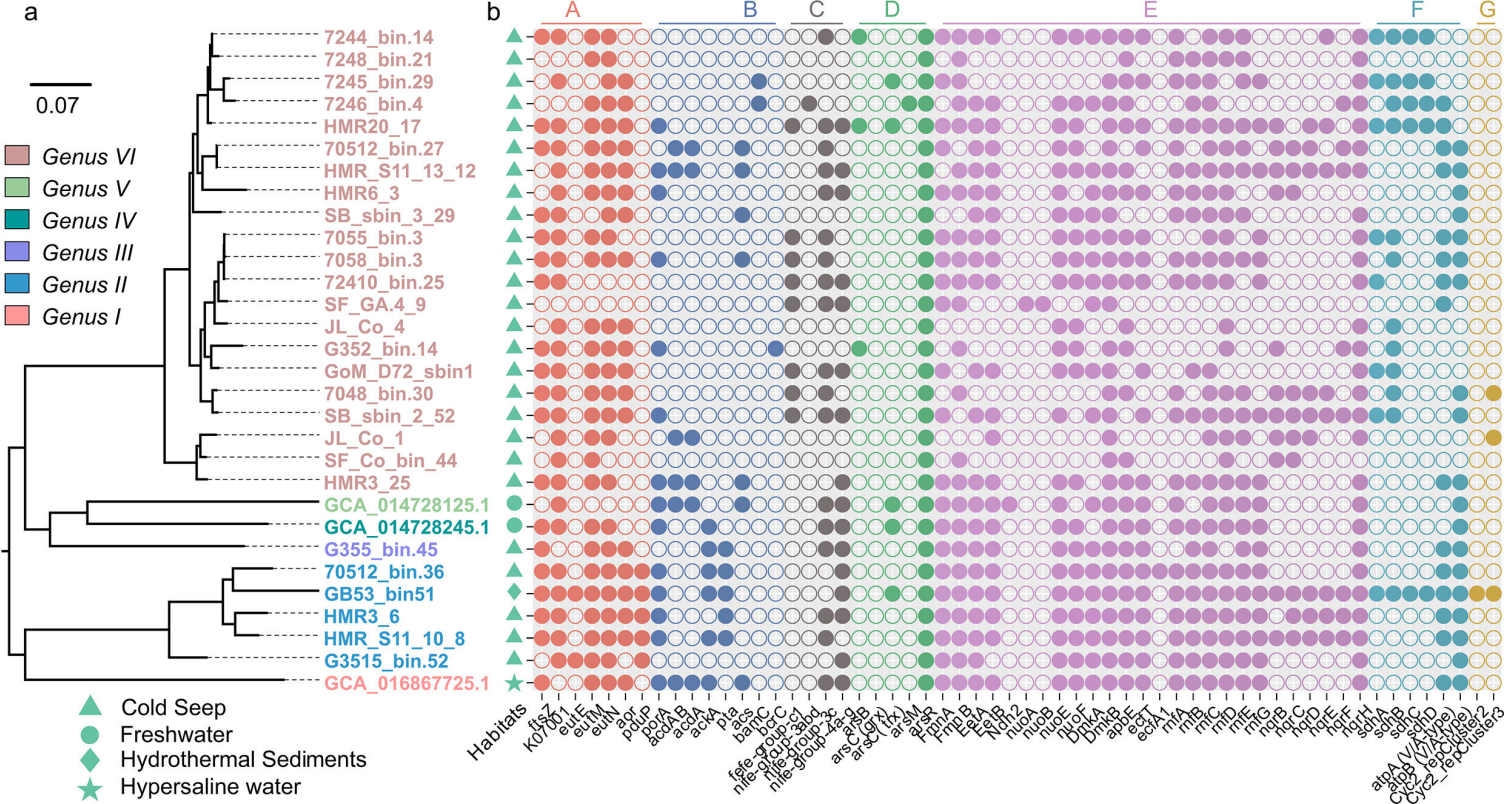
Phylogenetic distribution of habitats and key genes within *Candidatus* Effluviviacota. The phylogeny for MAGs representing genus-level lineages (I–VI) of *Candidatus* Effluviviaceae (phylum *Candidatus* Effluviviacota) includes *Candidatus* Effluvivivax and *Candidatus* Effluvibates as groups II and VI, respectively (a). In the environment column (b), different symbol shapes represent the different source habitats of the reconstructed MAGs. Color-coded segments in the gene matrix, labeled A–G, represent the key genes related to bacterial microcompartments (BMCs), carbohydrate and hydrocarbon metabolism, hydrogen metabolism, arsenate reduction detoxification mechanisms, extracellular electron transfer (EET), oxidative phosphorylation, and iron cycling (b).

ANI and AAI are reliable indicators for species circumscription, genomic discreteness, and functional gene content similarity, respectively. Based on ANI (Table S4), AAI (Table S5), and 16S rRNA gene phylogeny approaches (Table S13), all 30 MAGs in the *Ca.* Effluviviacota belong to the same family and shared at least 45% AAI similarity. The MAGs are divided into six distinct lineages at the genus level, I–VI (Fig. 2a), with 45%–65% shared AAI similarity (7). Within genus II, the MAG with the highest degree of completeness, HMR3_6 (Table S3), clustered with four closely related MAGs (GB53_bin.51, HMR_S11_10_8, 70512_bin.36, and G3515_bin.52). For these MAGs, we propose the genus-level candidatus taxon “*Candidatus* Effluvivivax,” in reference to their affinity for diverse seepage habitats. In addition, for genus VI, which contains the largest number of MAGs, we propose the genus-level candidatus taxon “*Candidatus* Effluvibates,” based on the observation that all known MAGs within this group inhabit cold seep sediments. Lineages I, III, IV, and V have not been named in this study because they are insufficiently covered by MAGs and require further research and recovery of high-quality MAGs before they can be further named. Thus, the family-level *Candidatus* Effluviviaceae includes *Ca*. Effluvivivax, *Ca*. Effluvibates, and related but currently unnamed genus-level lineages (Fig. 2a). It is noteworthy that the GC content of *Ca*. Effluvivivax is relatively low, ranging from approximately 59% to 61%, whereas the GC content of *Ca*. Effluvibates is comparatively high, ranging from approximately 63% to 65%. This disparity in GC content suggests lineage-specific evolutionary adaptation to environmental variations, independent of geographical location.

### Distribution of *Ca*. Effluviviacota in nature

**Fig 3:**
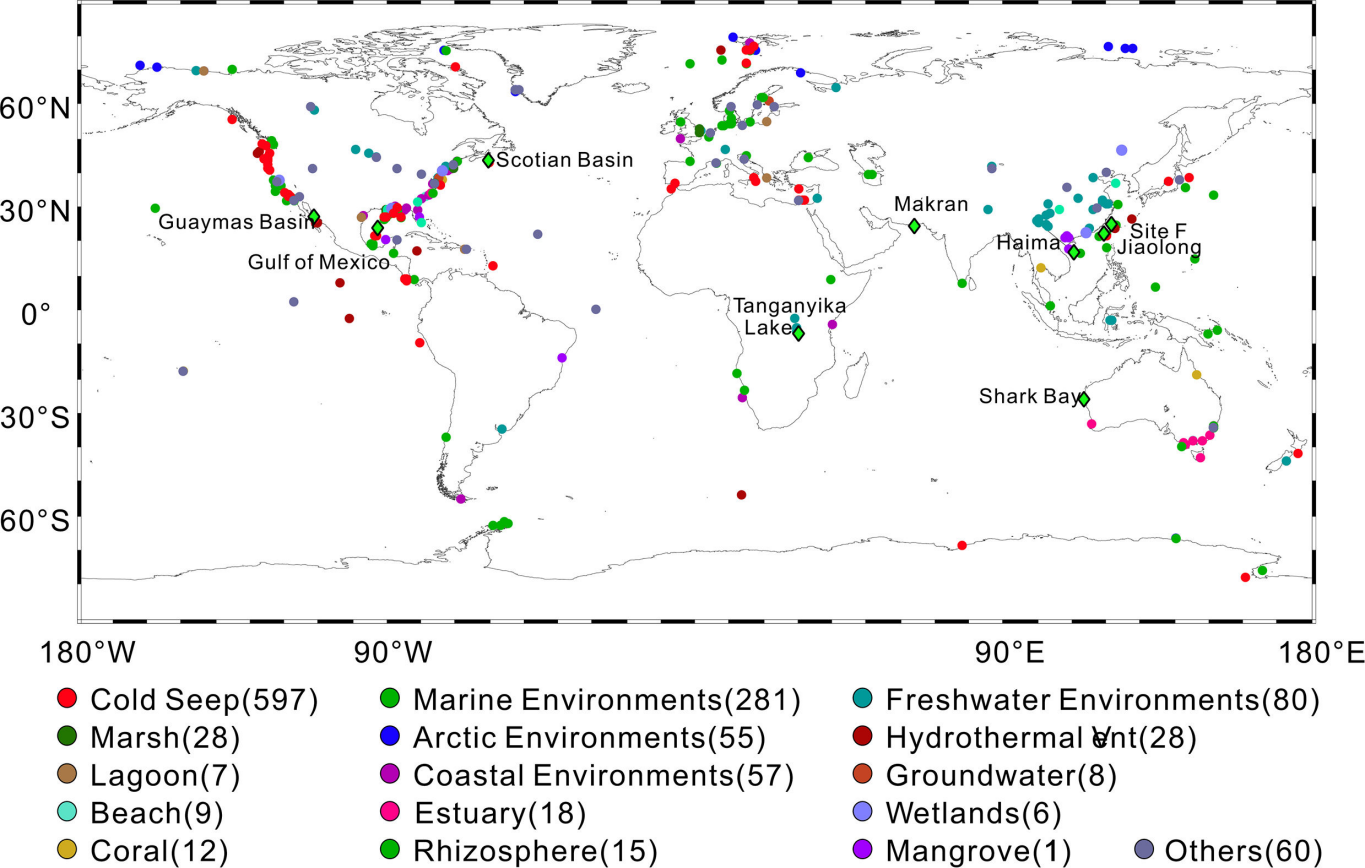
Global occurrences of *Candidatus* Effluviviacota genomes and 16S rRNA gene sequences. The dots in different colors represent the geographic localization from which *Candidatus* Effluviviacota sequences were obtained from MAPseq based on level 90. The numbers in parentheses represent the number of 16S rRNA gene sequences queried in their corresponding habitat (Table S6). The green diamonds represent locations where we obtained metagenomic data. Ocean Data View was used to create a world map shape with geographic coordinates of *Candidatus* Effluviviacota sequences.

We obtained 1,272 16S rRNA gene sequences, of which 597 were found in different cold seeps around the world. Other environments include various freshwater and marine aquatic environments, arctic environments, coastal sediments, and hydrothermal vents (Fig. 3; Table S6). This distribution indicated that 16S rRNA gene sequences related to *Ca.* Effluviviacota come from diverse environments worldwide but likely prefer cold seep sediments as their primary ecological niche (Fig. 3). The geochemical gradients of the sampling sites in the Haima cold seep (20), Makran cold seep, and Guaymas Basin hydrothermal sediments (21) illustrate the environmental characteristics of an anoxic, methane-rich seep habitat for *Ca.* Effluviviacota (Fig. S1; Tables S1 and S2). The results of MAG relative abundance showed that *Ca.* Effluviviacota prefers seep niches characterized by high methane concentrations, with the highest relative abundance of 5.1% in the Haima cold seep sediments (Fig. S2). However, due to the limited number of MAGs, we cannot assess the global distribution characteristics of its abundance.

### Metabolic potential features of the genus *Ca*. Effluvivivax and *Ca*. Effluvibates

**Fig 4:**
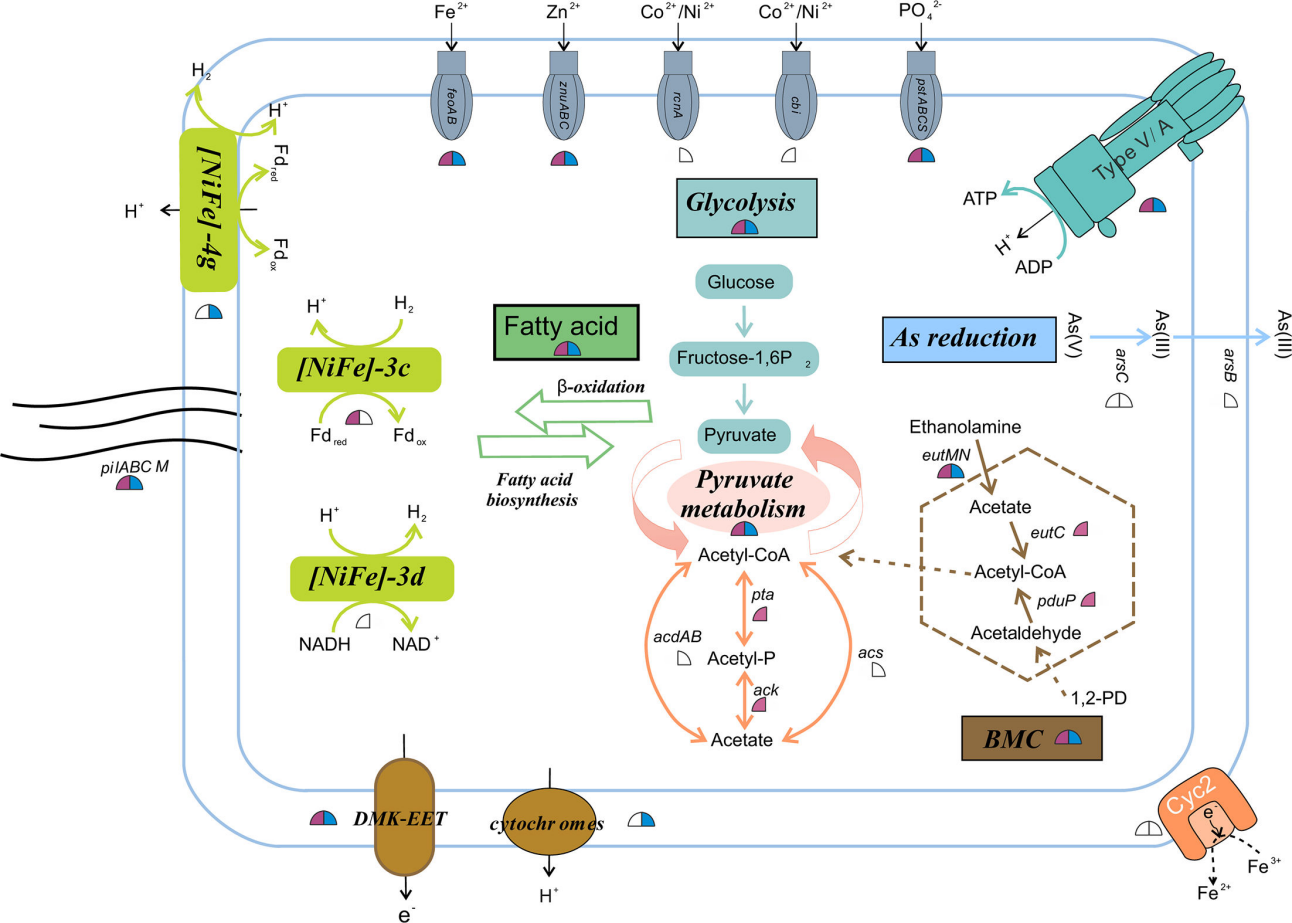
Overview of metabolic capabilities and membrane transport among *Candidatus* Effluvivivax and *Candidatus* Effluvibates. Different metabolic pathways are represented in different colors, with the direction of the arrow representing the direction of the metabolic process. The upper left quarter circles represent the genus *Ca*. Effluvivivax; the upper right quarter circles represent the genus *Ca*. Effluvibates. Filled shapes represent genes and pathways that occur in more than 50% of the number of MAGs; shapes for genes and pathways below this threshold are not filled. Abbreviations: DMK-EET: NUO-DMK-EET-FMN extracellular electron transfer complex; CoA, coenzyme A; Fd, ferredoxin; ATP, adenosine triphosphate; ADP, adenosine diphosphate.

The predicted proteins for *Ca*. Effluvivivax and *Ca*. Effluvibates (including the MAGs in Fig. 2a) were compared to their best matches in various databases and gene phylogenies to understand the structural and metabolic features of these bacteria (see Materials and Methods). It appears that members of *Ca.* Effluviviacota are strict anaerobes, as evidenced by the absence of respiratory cytochrome c oxidase component (complex IV) and the environmental preferences of these reconstituted MAGs (Fig. 2b; Tables S1 and S7). The genes rodA/mreB, which determine the rod-shaped morphology, and the genes responsible for flagellar assembly suggest that the cells are both rod-shaped and flagellated (Fig. 4; Table S8).

### Structural features

Structurally, members of *Ca*. Effluvivivax and *Ca*. Effluvibates are predicted to have a Gram-negative cell wall based on the possession of lipopolysaccharide biosynthesis-encoding genes (lpt) and lack of genes encoding the pentaglycine linkage of peptidoglycan (Table S8). The genomes of *Ca*. Effluvivivax and *Ca*. Effluvibates encode proteins with unique characteristics that may be linked to intricate cellular structures. For instance, the major cytoskeletal protein in the bacterial cytokinesis machine, cell division protein FtsZ (42), exhibits N-terminal extensions (NTEs), which are present in most *Ca*. Effluvivivax and *Ca*. Effluvibates MAGs (Fig. 2b and 4; Table S4). The NTE-possessing FtsZ protein may be able to bind to the membrane, but further investigation is required to confirm this.

It is noteworthy that NTEs in prokaryotes have only been found in enzymes that localize to the lumen of subcellular compartments known as BMCs (43, 44). BMCs are organelles enveloped with a protein shell that can promote specific metabolic processes by encapsulating and colocalizing enzymes with their substrates and cofactors (44–46). They can also serve as a protective enclosure for toxic or volatile intermediates, shielding the cytosol from toxic intermediates (46). BMCs allow for the encapsulation of a diverse range of metabolic pathways within the shell’s interior. This enhances reaction efficiency by spatially organizing pathway enzymes in proximity to metabolic partners, substrates, and cofactors (45, 47). However, the specific functions of BMCs vary, depending on the genes adjacent to the shell protein genes within the BMC gene loci (47).

The function of microcompartments shows significant differences between *Ca*. Effluvivivax and *Ca*. Effluvibates. The presence of coenzyme A-acylating propionaldehyde dehydrogenase (PduP) in all *Ca*. Effluvivivax MAGs suggests that the putative *Ca*. Effluvivivax BMC may sequester metabolically generated, toxic aldehydes as has been proposed for catabolic BMCs in other organisms (45, 48). However, the lack of several other key Pdu genes required for 1,2-propanediol (1,2-PD) catabolism suggests that this specific substrate is probably not utilized by *Ca*. Effluvivivax. Some *Ca*. Effluvivivax MAGs encode the potential for ethanolamine metabolism within the ethanolamine utilization (EUT) microcompartment (Table S8), which appears to be closely related to the propanediol utilization (PDU) microcompartment in terms of encoded enzymes and chemical reactions (44). The dehydration of the three-carbon compound 1,2-PD generates propionaldehyde in the PDU microcompartment, while the deamination of the two-carbon compound ethanolamine leads to acetaldehyde in the EUT microcompartment (49). The specific functions attributed to BMC structures, as outlined above, are conspicuously absent in *Ca*. Effluvibates but not in *Ca*. Effluvivivax. The divergence in BMC function may be linked to different habitat preferences; *Ca*. Effluvivivax is found in both cold seeps and hydrothermal sediments, whereas members of *Ca*. Effluvibates are restricted to only cold seeps.

### Carbohydrate and hydrocarbon metabolism

While genes for carbon fixation pathways were not detected (Tables S7 and S8), all members of *Ca*. Effluvivivax and *Ca*. Effluvibates encode the complete Embden–Meyerhof glycolysis pathway (Fig. 2b and 4; Tables S7 and S8). The terminal reaction step of glycolysis is the oxidation of pyruvate to acetate (50).

Acetate is considered as an essential intermediate in microbial carbohydrate utilization, fermentation, and respiratory pathways in cold seep and hydrothermal sediments (51, 52). The most abundant MAGs in benthic seep sediments are generally acetogenic heterotrophs (12), which play a quantitatively important role in organic carbon cycling in the marine deep biosphere (53, 54). *Ca*. Effluvibates possessed genes encoding phosphate acetyltransferase (pta) and acetate kinase (ackA) (Fig. 2b and 4; Table S8), which catalyze acetogenesis from acetyl-CoA and simultaneously produce ATP (54). In addition, acetate could also be produced through the intermediate acetyladenylate via acetyl-CoA synthetase (acs) or acetate-CoA ligase in *Ca*. Effluvibates (Fig. 4). Unexpectedly, 70512_bin.27, HMR_S11_13_12, and HMR3_25, which represent *Ca*. Effluvivivax, possessed another (ADP-forming) type of acetyl-CoA synthetase (acdAB) (Fig. 2b). This enzyme is mainly distributed in archaeal genomes (54), until recently when several acdAB homologs were found in bacterial genomes (55, 56). The reason for divergent acetate metabolic pathways between *Ca*. Effluvivivax and *Ca*. Effluvibates remains unclear and deserves further investigation. In any case, acetogenesis is widespread in *Ca.* Effluviviacota, and these widespread bacteria could potentially serve as a source of acetate for other microorganisms, including acetoclastic methanogens, in global cold seep environments (19).

Glycoside hydrolases (GHs) (57) and polysaccharide lyases (PLs) (58) appear to be the most abundant CAZymes within *Ca.* Effluvivivax and *Ca*. Effluvibates (Table S10). Among these CAZymes, both *Ca*. Effluvivivax and *Ca*. Effluvibates feature a variety of GH families capable of degrading cellulose, hemicellulose, starch, and xylan (via hydrolytic enzymes that convert polysaccharides into monosaccharides), such as GH003, GH013, GH023, GH057, GH097, and GH109, as well as PL001, PL012, and PL015 (Table S10). In addition, a variety of peptidases are also abundant in *Ca*. Effluvivivax and *Ca*. Effluvibates (Table S11). These findings align with the abundance of organic matter found in cold seeps and the Guaymas Basin.

Our results also provide evidence that aromatic compounds can be degraded anaerobically via the central benzoyl-CoA degradation pathway found in some MAGs (Fig. 2B; Tables S7 and S8). Benzoyl-CoA can be reduced to cyclohex-1,5-diene-1-carboxyl-CoA by either the Class I ATP-dependent benzoyl-CoA reductase pathway (bcr genes) or the Class II ATP-independent reductase (bam genes) pathway (59). We identified the bcrC gene for the Class I pathways only in 7244_bin.14 (*Ca*. Effluvibates) and the bamC gene for the Class II pathway only in 7245_bin.29 and 7246_bin.4 (*Ca*. Effluvibates). Notably, these samples were all obtained from deep sediments characterized by high methane concentrations within and below the sulfate–methane transition zone (SMTZ) (Fig. S1). Although only part of the central benzoyl-CoA degradation pathway was detected in *Ca*. Effluvibates, these bacteria may have a competitive advantage in utilizing aromatic compounds, potentially outcompeting sulfate-reducing bacteria specialized in aromatic degradation (21, 60, 61), once sulfate becomes limiting in these samples.

Members of *Ca.* Effluviviacota do not participate in the oxidation of methane or alkanes, which are abundant in cold seeps (8), but they participate in the anaerobic degradation of organic substrates at cold seeps (Tables S7 and S8), which may be produced by chemosynthetic or methane-oxidizing organisms. These heterotrophic abilities would give *Ca.* Effluviviacota a trophic position analogous to that of secondary hydrocarbon utilizers, which do not degrade seep hydrocarbons directly but utilize hydrocarbon-derived biomass (62). Specializing in this ecological niche may explain the wide distribution and abundance of *Ca.* Effluviviacota in cold seep sediments and suggest their particular role in the global marine carbon cycle.

### Hydrogen metabolism

H_2_ metabolism contributes to the growth and survival of microorganisms, including chemotrophs and phototrophs, lithotrophs and heterotrophs, aerobes and anaerobes, and mesophiles and extremophiles alike (63, 64). It has been widely proposed that H_2_ is the primordial electron donor that has provided metabolic energy already during early microbial evolution (65). The key enzyme of microbial hydrogen turnover, hydrogenases, is divided into distinct subgroups based on their biochemical characteristics (e.g., directivity, affinity, redox partner, and localization) and physiological functions (e.g., respiration, fermentation, bifurcation, and sensing) (63, 64). Several hydrogenase-encoding genes were identified in the genomes of *Ca*. Effluvivivax and *Ca*. Effluvibates (Fig. 2b; Tables S7 and S8).

*Ca*. Effluvivivax contained genes for methanogen-specific [FeFe]-hydrogenase (64), which is typically associated with proton reduction (Fig. 2b and 4). Hydrogenase genes of the [NiFe] classes, in particular group 3, were widely distributed within the *Ca.* Effluviviacota (Fig. 2b and 4; Table S9). Since [NiFe] group 3d hydrogenases catalyze the production of H_2_ with NADPH or NAD(P)H as the electron donor during fermentation (66), we infer that *Ca*. Effluvivivax might be able to catalyze fermentative NADH-dependent production of H_2_ (Fig. 2b and 4). Group 4 membrane-bound [NiFe]-hydrogenases were widely distributed in *Ca*. Effluvivivax and *Ca*. Effluvibates (Fig. 2b and 4). These hydrogenases are defined as the H_2_-evolving energy-conserving membrane-associated hydrogenases (66) and play an important role in conserving energy by establishing ion gradients across membranes that can be used to generate ATP (642). Recycling of electron carriers would further be achieved by the cytoplasmic [NiFe]-hydrogenase group 3c (Fig. 2b and 4). In group 3, the dimeric hydrogenase module is associated with other subunits able to bind soluble cofactors, such as cofactor 420 (F420, 8-hydroxy-5-deaza flavin), NAD (1,4-dihydroxy-2-naphthoate), or NADP. They are termed bidirectional because, physiologically, they function reversibly and can, thus, reoxidize the cofactors under anaerobic conditions by using the protons of water as electron acceptors (66).

### Extracellular electron transfer

EET describes a class of microbial activities that result in the transfer of electrons from the cytosol to the outside of the cell and often function in heterotrophic respiration (67, 68). EET is mainly realized by an electron transport chain of redox-active proteins within the cell envelope and on the outer cell membrane, using electron shuttle mechanisms such as flavins to transfer electrons from inside the cell to the external environment (69). The advantage of electron shuttles is that they allow microorganisms to circumvent the need for direct contact with extracellular electron acceptors (69, 70).

**Fig 5:**
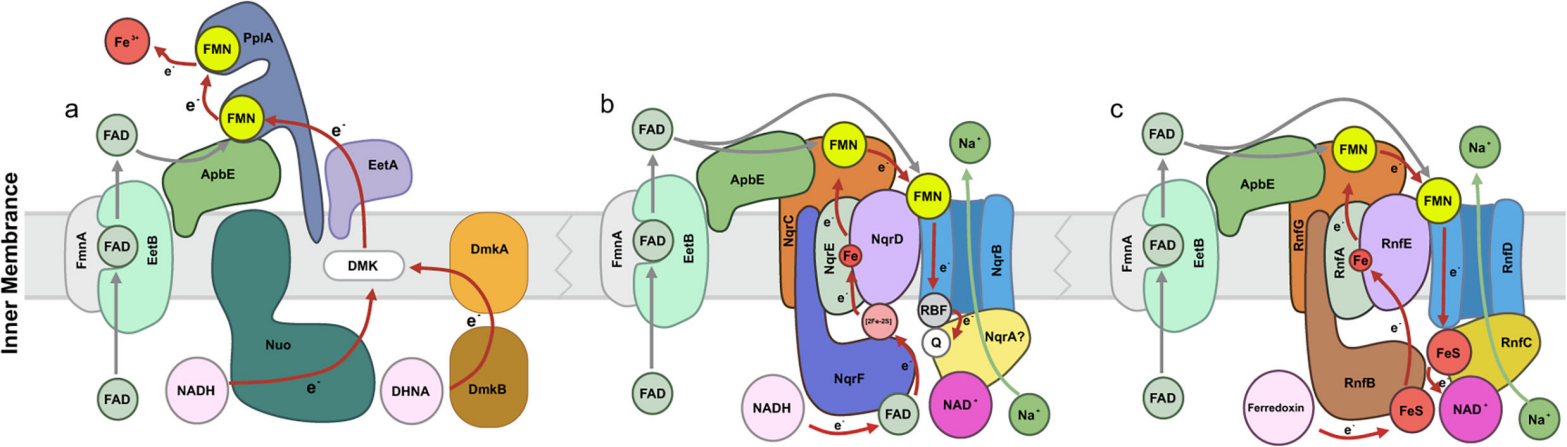
Overview of EET apparatus in *Ca*. Effluviviacota. A surface-associated flavoprotein establishes the extracellular component of the EET apparatus. Red arrows represent electron transfer, and green arrows represent flavin transfer. The proposed mechanism of flavin export for *Ca*. Effluviviacota flavinylation and EET activity are shown in the panel on the left (a). Proposed models of flavin export in previously identified flavinylation-associated Nqr complexes, Rnf complexes, and speculative electron transfer pathways are shown in the central and right panels (b, c).

EET-related genes encoding orthologs for complex I (NADH-quinone oxidoreductase; Nuo) components, demethylmenaquinone-synthesizing enzymes (DmkAB), uncharacterized membrane proteins (EetAB), and a series of proteins required for the secretion of flavin cofactors used by terminal reductases (e.g., FmnAB) (67) are indeed prevalent in *Ca.* Effluviviacota (Fig. 5a; Table S8). Homologous genes are present in several related members of the Firmicutes and have been implicated in similar EET activities in several bacteria (71–73). The Nuo complex can transfer electrons from NAD to DMK (demethylmenaquinones), then transfer to FMN (flavin mononucleotide) groups on PplA or free flavin shuttles—possibly with involvement from uncharacterized membrane proteins in the EET locus and uncharacterized membrane proteins (EetA and EetB)—and ultimately to a terminal electron acceptor. In this context, the NUO-DMK-EET-FMN electron transport pathway is named based on the abbreviations of enzymes necessary for this electron transport pathway. Although we did not find all the genes associated with complete complex I in *Ca*. Effluvibates and *Ca*. Effluvivivax, we observed the presence of group 4 membrane-bound [NiFe]-hydrogenases and mrp-type antiporters, which are structurally similar to the membrane domain of respiratory complex I (74). These findings suggest a potential role for these genes in the metabolic processes of *Ca*. Effluvibates and *Ca*. Effluvivivax. Specifically, we identified all mrpBCDEFG genes in two MAGs (7055_bin.3 and 7058_bin.3) of *Ca*. Effluvibates, while group 4 membrane-bound [NiFe]-hydrogenases were detected in the majority of *Ca*. Effluvibates and *Ca*. Effluvivivax genomes (Fig. 2b; Table S9).

In addition to the Nuo complex, the Rnf and Nqr complexes also have the potential to transfer electrons. In prokaryotes, many extracytosolic flavoproteins are posttranslationally linked to their flavin cofactors (flavinylated) through the action of the ApbE enzyme (75). Nitrogen fixation (Rnf) and NADH:quinone oxidoreductase (Nqr) are prominent multisubunit complexes with ApbE-flavinylated subunits that possess important roles in diverse bacteria and energy metabolisms (76, 77). However, Rnf and Nqr complexes are not universal in all *Ca.* Effluviviacota but occur in some members of *Ca*. Effluvivivax and *Ca*. Effluvibates (Fig. 2b, 5b and c). Overall, this pattern of gene distribution underscores the heterogeneity within *Ca.* Effluviviacota and suggests varying roles in energy metabolism among different genera of this phylum. Further research is needed to explore the functional implications of these findings and the potential interactions between these genes and other metabolic pathways. The discovery of flavin-based EET mechanisms in *Ca.* Effluviviacota represents the first documented occurrence of this mechanism outside the Gram-positive bacteria and opens up new possibilities for extracellular electron transfer in Gram-negative bacteria.

Further investigations, focusing on the EetA and EetB proteins pivotal in electron and flavin transfer pathways, have revealed the widespread presence of EetAB across more than half of the *Ca.* Effluviviacota population (Fig. 2b), along with its extensive distribution among various bacterial taxa, particularly within *Listeria* (Fig. S8). Consequently, we propose that the observed flavin-based EET genes in *Ca.* Effluviviacota primarily result from horizontal gene transfer events with other microbial lineages; yet, determining the evolutionary origin of EetAB will require an extended database and further investigations.

Additional possible mechanisms for extracellular electron transport may involve outer-membrane cytochromes. We detected the presence of the multiheme cytochrome superfamily in almost all MAGs of *Ca*. Effluvivivax and *Ca*. Effluvibates (Table S12). Fourteen of the analyzed genomes contained at least one multiheme cytochrome with a signal peptide indicating export out of the cytoplasm and a possible role in extracellular electron transport, which occurs predominantly but not exclusively via the general secretory or Sec pathway (Fig. 4; Table S12). Notably, the multiheme cytochrome with signal peptide, which is prevalent in *Ca*. Effluvivivax, has exclusively been detected within *Ca*. Effluvibates from the Guaymas Basin hydrothermal sediment (Table S12). Multiheme cytochrome electron transfer proteins have been shown to support electron exchange between cells and the extracellular environment (69, 78). These EET pathways facilitate microbial respiration with extracellular metals and minerals (69) and extend the potential of *Ca.* Effluviviacota to utilize metal ions as electron acceptors. Additionally, we observed the presence of the cyc2 gene in some MAGs of *Ca*. Effluvivivax and *Ca*. Effluvibates (Fig. 4), likely functioning as a membrane-bound catalyst for redox reactions with metals (79).

Overall, the electron transport mechanism suggests that *Ca.* Effluviviacota has the capability to utilize metal oxyhydroxides as electron acceptors (68–70). Metal reduction is a desirable capability in seep sediments, as previous studies have emphasized the prevalence of metallic elements, including Fe(III) and Mn(IV), within and beneath the SMTZ (80, 81).

### Arsenate reduction detoxification mechanisms

Arsenic (As) is common in cold seep and hydrothermal sediments (82, 83). Both *Ca*. Effluvivivax and *Ca*. Effluvibates contain the arsenic detoxification system that catalyzes the reduction of arsenate (AsO43^−^) to arsenite (As(OH)_3_) by intracellular arsenate reductase (ArsC) (84). Methylation and cytoplasmic As(V) reduction were the predominant arsenate detoxification mechanisms employed by cold seep and hydrothermal microbiomes (85). The exact molecular mechanism of As(V) reduction in *Ca*. Effluviviacota is still unknown.

### Conclusions

We introduce a novel bacterial phylum, *Ca.* Effluviviacota, formerly known as VGIX01. *Ca.* Effluviviacota is predominantly found in seep niches characterized by elevated methane concentrations, and its MAGs account for a notable portion of the cold seep ecosystem, ranging from 1% to 5.1% of all MAGs recovered from these habitats. Members of *Ca.* Effluviviacota possess multiple adaptive features, including distinctive extracellular electron transport mechanisms, arsenic detoxification pathways, bacterial microcompartments, and motility, contributing to their global prevalence in cold seep ecosystems. The identification of the NUO-DMK-EET-FMN electron transport complex and its homologous genes within *Ca.* Effluviviacota suggests an extension of this electron transport pathway from Gram-positive bacteria to *Ca.* Effluviviacota. This observation supports the hypothesis that the flavin-based EET genes observed in *Ca.* Effluviviacota have primarily originated from horizontal gene transfer events with other microbial lineages. *Ca.* Effluviviacota is not directly involved in methane or alkane oxidation and, therefore, does not belong to the group of bacteria responsible for the direct conversion of fossil organic carbon into biomass. By participating in the heterotrophic degradation of organic matter that is produced either chemosynthetically or by oxidation and assimilation of methane and hydrocarbons, *Ca.* Effluviviacota occupies a widely distributed ecological niche in the trophic cascade that remineralizes seep-derived biomass at hydrocarbon seeps.

#### *Candidatus* Effluvivivax

##### Etymology

Effuvivivax is derived from effluvium (Latin) [invisible exhalation or vapor (including unpleasant smell)] and vivax (Latin) tenacious of life [haimensis (Latin) pertaining to the Haima seep area].

##### Locality

Effuvivivax is located in the Haima Seep Area, South China Sea, and Guaymas Basin, Gulf of California.

##### Description

*Candidatus* Effluvivivax is a mesophilic, heterotrophic, anaerobic bacterium that is capable of fermenting low-molecular-weight organic substrates (acetate) and of carbon, hydrogen, and metal cycling. Members of *Ca*. Effuvivivax have the ability to metabolize toxic aldehydes within its catabolic bacterial microcompartment, which separates this group from *Candidatus* Effluvibates.

### *Candidatus* Effluvibates

#### Etymology

Effluvibates is derived from effluvium (Latin) [invisible exhalation or vapor (including unpleasant smell)] and batos (Latin) meaning accessible and viable and is used in zoology in reference to habitat.

#### Locality

Effluvibates is located in the Haima Seep Area, South China Sea; Jiaolong cold seep, South China Sea; Site F cold seep, South China Sea; Makran cold seep, Indian Ocean; Scotian Basin, Eastern Canada; and Gulf of Mexico.

#### Description

*Candidatus* Effluvibates is a mesophilic, heterotrophic, anaerobic bacterium that is capable of fermenting low-molecular-weight organic substrates (acetate) and of carbon, hydrogen, and metal cycling. *Ca*. Effluvibates have the genome content to participate in the biogeochemical cycling and detoxification of As.

At present, *Ca.* Effluviviacota are best documented by reconstructed MAGs from methane- and sulfide-rich cold seep sediments and similar benthic habitats.

## Data Availability

All sample information and metagenome-assembled genomes in this study are available at NCBI under BioProject ID PRJNA1050653. The freshwater MAG from Lake Tanganyika can be accessed on NCBI BioProject ID PRJNA523022; Shark Bay MAGs are deposited under BioProject “Novel microbial dark matter shaping Shark Bay microbialites” (PRJNA561032).

## SUPPLEMENTAL MATERIAL

Supplemental Material mbio.00992-24-s0001.pdf Figures S1 to S8 and legends for Tables S1 to S13.

Supplemental Tables mbio.00992-24-s0002.xlsx Tables S1-S13.

ASM does not own the copyrights to Supplemental Material that may be linked to, or accessed through, an article. The authors have granted ASM a non-exclusive, world-wide license to publish the Supplemental Material files. Please contact the corresponding author directly for reuse.
