# Supplementary material for: Genomic characterization of the bacterial phylum Candidatus Effluviviacota, a cosmopolitan member of the global seep microbiome
Source: mBio. 2024 Jul 9;15(8):e00992-24. doi: 10.1128/mbio.00992-24 (PMC11323493; doi:10.1128/mbio.00992-24)
Supplement: Supplemental Material — Figures S1 to S8 and legends for Tables S1 to S13. [file mbio.00992-24-s0001.pdf]

Supplementary Materials

Genomic characterization of the Bacterial Phylum *Candidatus* Effluviviacota, a cosmopolitan member of the global seep microbiome

Lei Su[1,2], Ian P. G. Marshall[2], Andreas P. Teske[3], Huiqiang Yao[4], Jiangtao Li[1*]

1 State Key Laboratory of Marine Geology, Tongji University, Shanghai 200092, China.

2 Center for Electromicrobiology (CEM), Section for Microbiology, Department of Biology, Aarhus University, Aarhus C, Denmark.

3 Department of Earth, Marine and Environmental Sciences, University of North Carolina at Chapel Hill, Chapel Hill, NC 27599, USA.

4 MLR Key Laboratory of Marine Mineral Resources, Guangzhou Marine Geological Survey, Guangzhou 510075, China.

[*]Corresponding author: jtli@tongji.edu.cn

The PDF file includes:

Figure S1 to S8

Legends for Table S1 to S13

Supplementary Figures

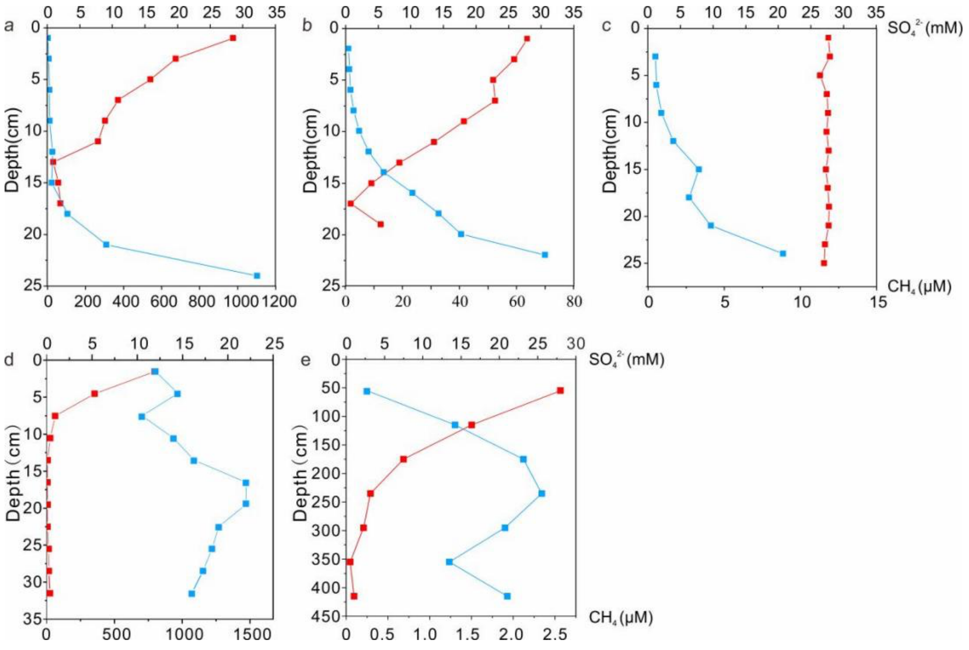

Figure S1 Vertical profiles of methane and sulfate concentrations in Haima cold seep sediments of the South China Sea (a, station 724; b, station 705; c, station 704), Guaymas Basin hydrothermal sediments (d, core 4573-23) and Makran cold seep sediments of the Indian Ocean (e, G35 station).

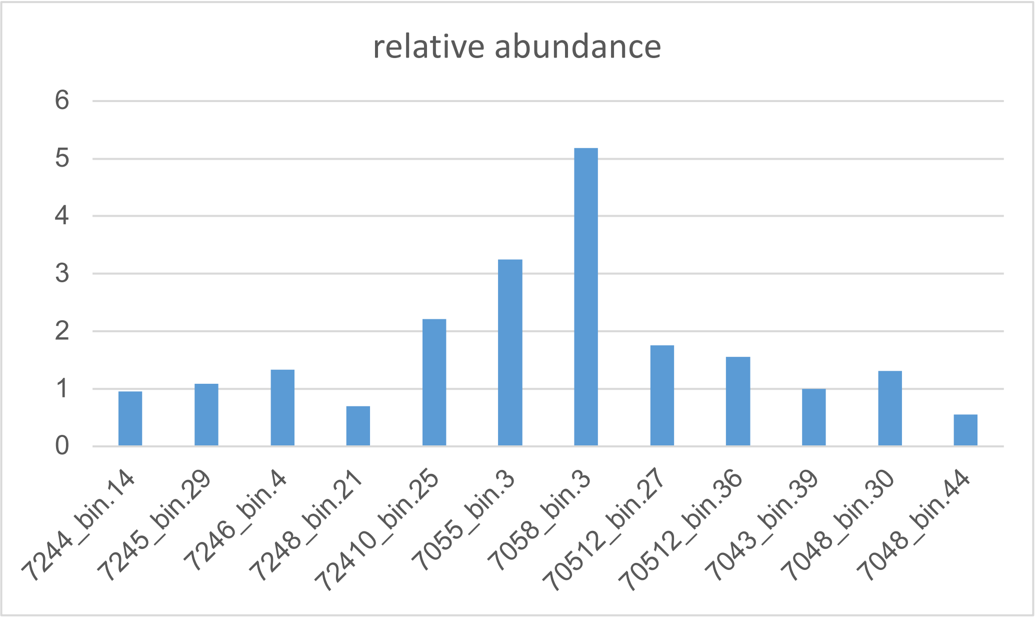

Figure S2 The relative abundance of *Ca.* Effluviviacota among all Metagenome-Assembled Genomes (MAGs) in Haima cold seep sediments.

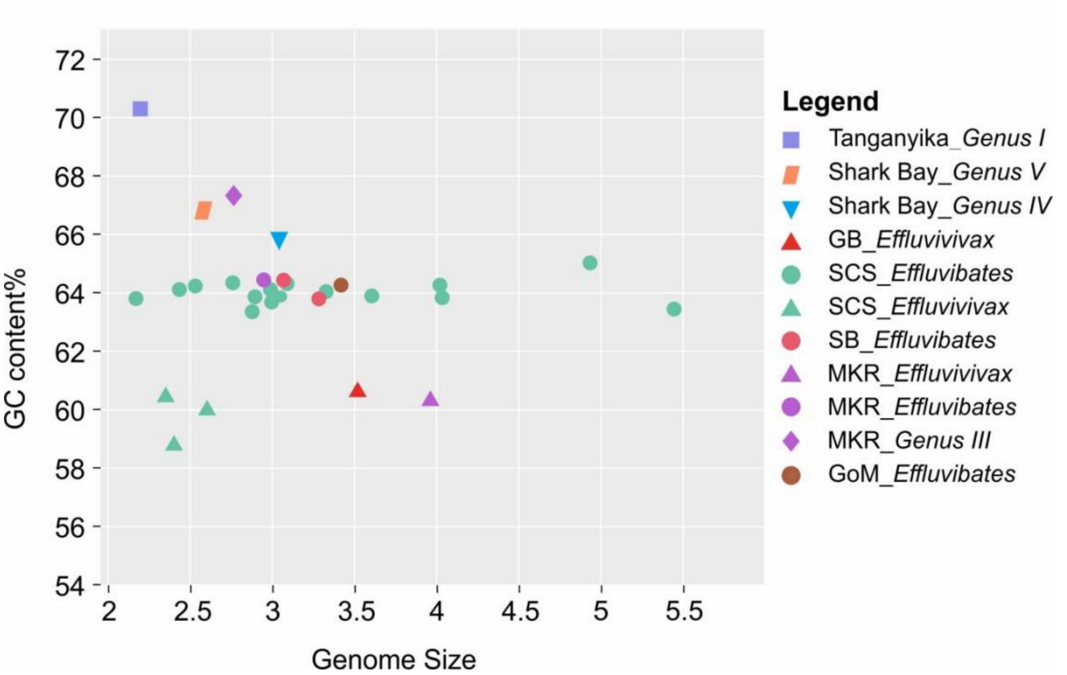

Figure S3 Relationship between true genome size, based on degrees of completeness and contamination, and GC content of 30 MAGs in this study.

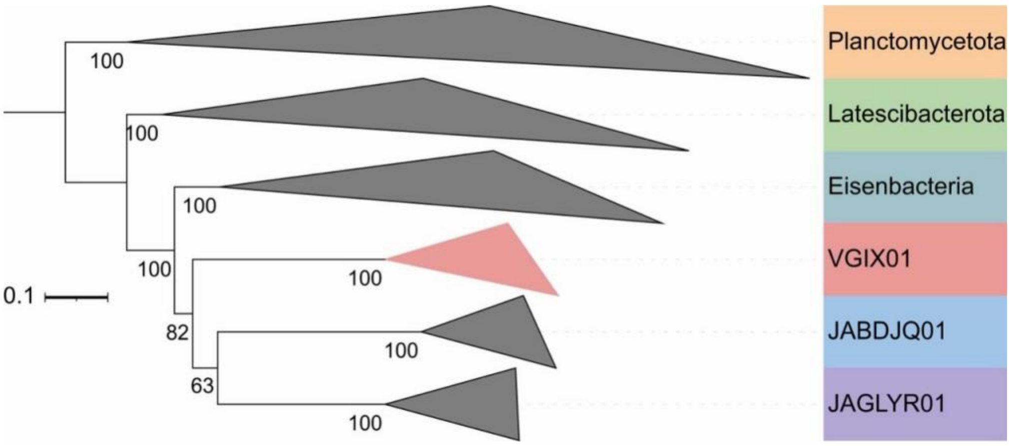

Figure S4 Maximum-likelihood phylogenetic tree of VGIX01 and references based on the assembled metagenomes. The genome tree was inferred using IQ-TREE (v.2.1.4; https://github.com/iqtree/iqtree2) with the parameters -m MFP -b 100 -T 10 and the best-fit evolutionary model (LG+F+R10). Bootstrap supports for nodes in the branching pattern are shown as percentages.

.

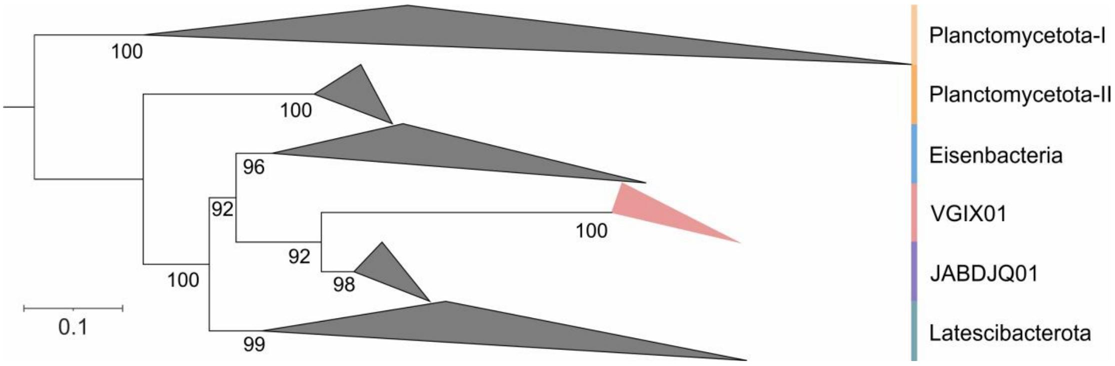

Figure S5 Maximum-likelihood phylogenetic tree of VGIX01 and references based on the 16S rRNA genes. The phylogeny was inferred using IQ-TREE with the best-fit evolutionary model (TIM3+F+I+G4) and 1,000 bootstrap replicates. Bootstrap supports for nodes in the branching pattern are shown as percentages.

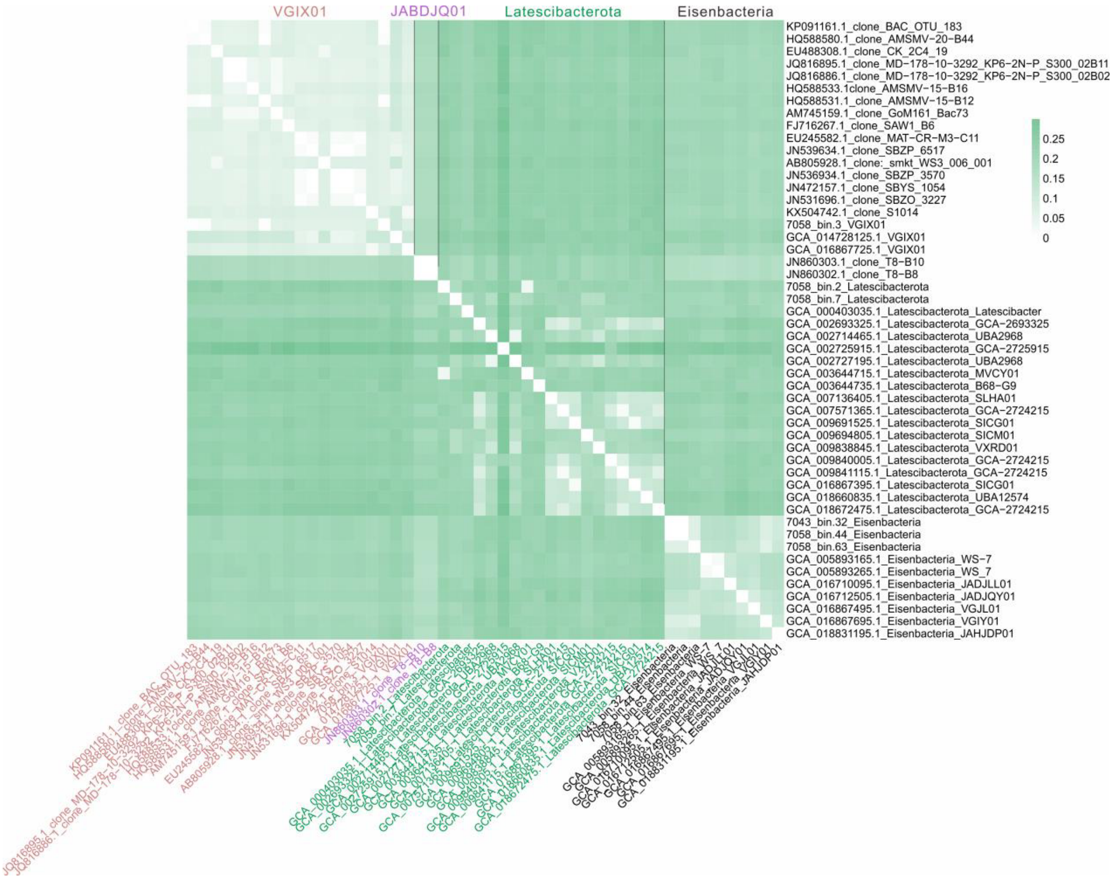

Figure S6 Pairwise nucleotide divergence in full-length 16S rRNA gene sequences between phyla VGIX01, JABDJQ01, *Eisenbacteria*, and *Latescibacterota*.

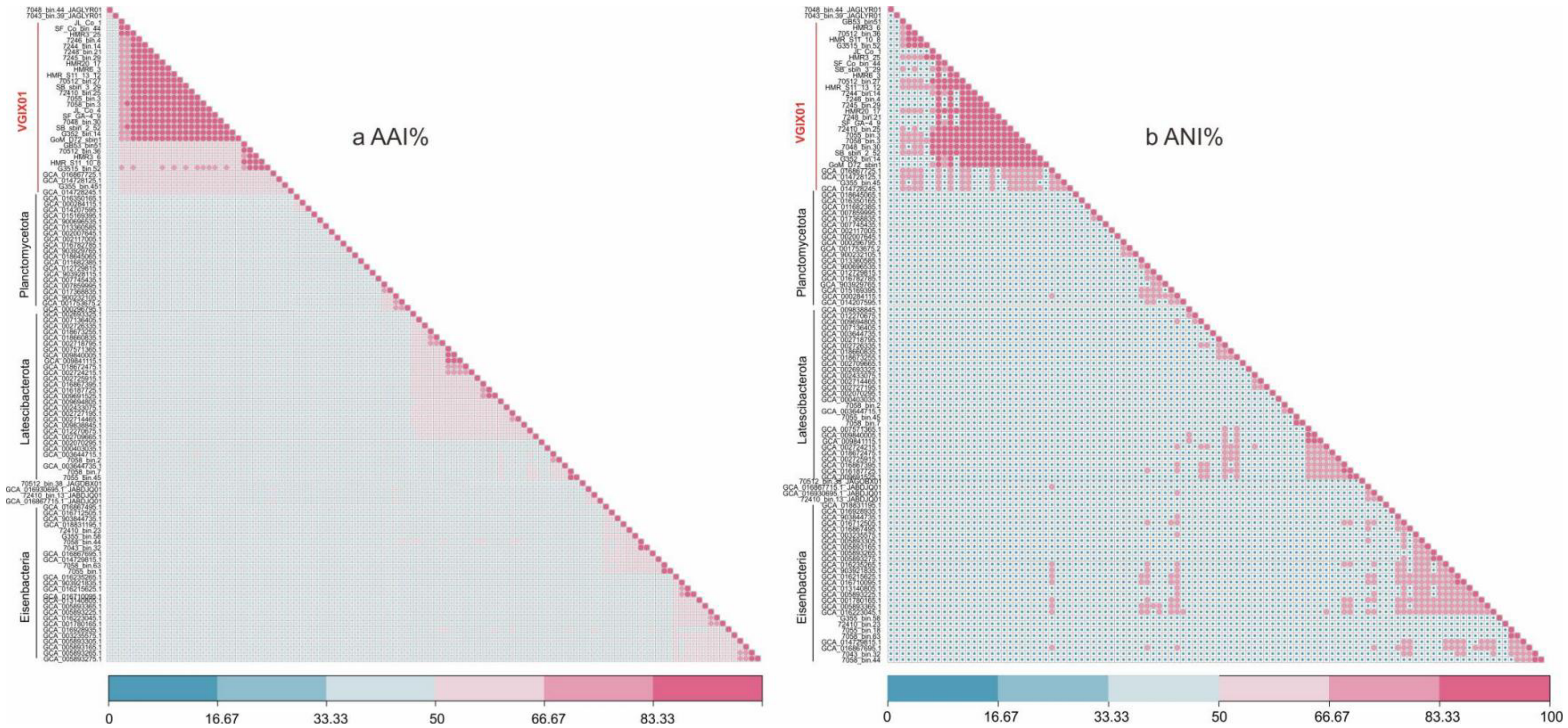

Figure S7 Pairwise genome-aggregate amino acid identity (AAI) (a) and average nucleotide identity (ANI) (b) of MAGs based on compareM and fastANI.

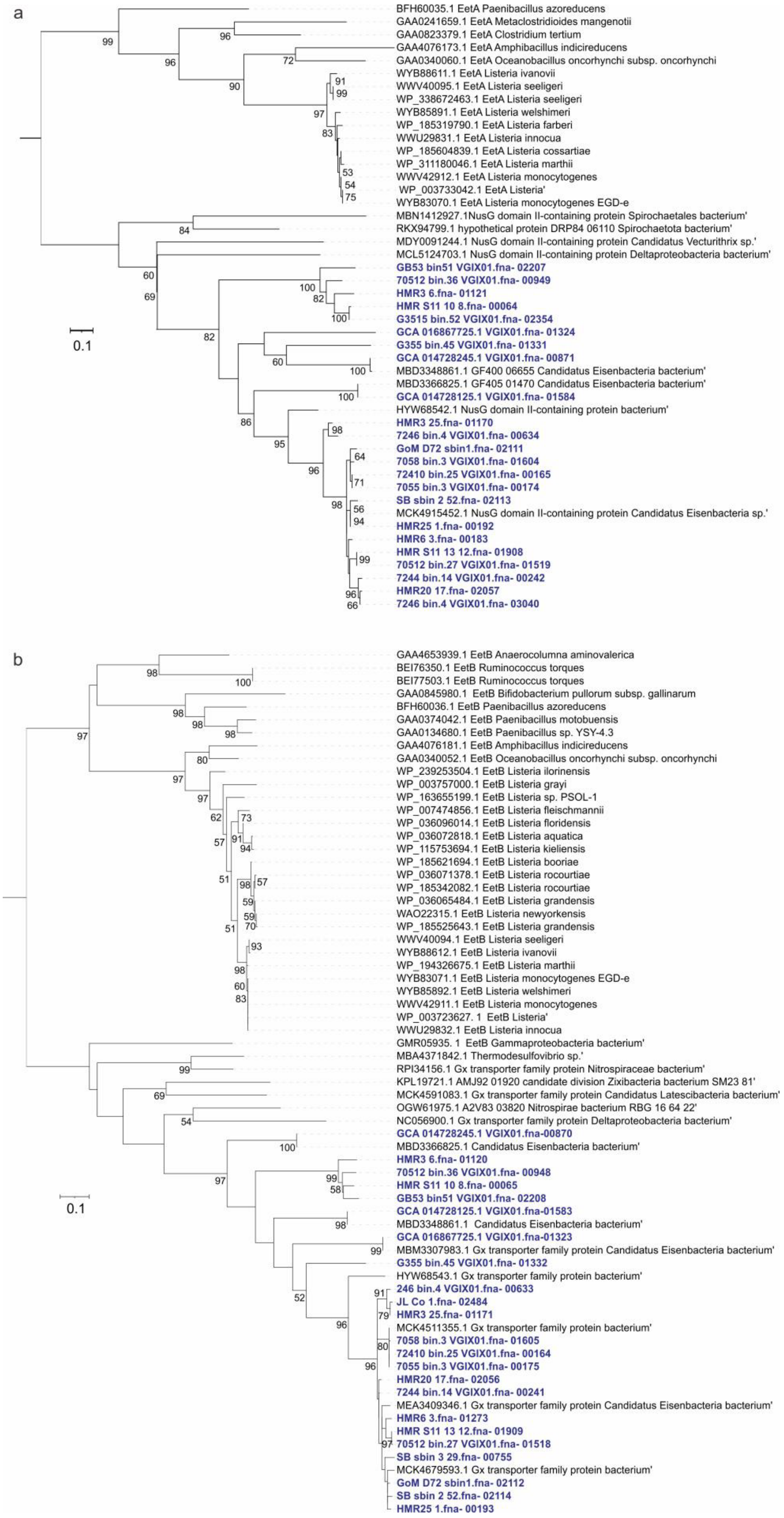

Figure S8 Phylogenetic tree of EetA (a) and EetB (b) protein sequences in *Candidatus* Effluvivacota and their reference sequences. It was inferred using MEGAX with the 1,000 bootstrap replicates.

Legends for supplementary tables

Table S1. Sampling and sequencing information for metagenomic data from NCBI and Figshare.

Table S2. Metadata for sampling sites.

Table S3. Basic information for 30 MAGs analysed in this study.

Table S4. Genome-aggregate average nucleotide identity (ANI) of MAGs, based on fastANI.

Table S5. Average amino acid identity (AAI) of MAGs, based on compareM.

Table S6. Metadata for VGIX01 16S gene sequences, obtained from MAPseq based on level 90.

Table S7. Functional annotations for key pathways using METABOLIC.

Table S8. Functional annotations for key pathways using KofamScan.

Table S9. Key genes for *Ca*. Effluvivivax and *Ca*. Effluvibate srepresented in Figure 4

Table S10. Overview of carbohydrate-active enzymes (CAZymes) results annotated using dbCAN2.

Table S11. Overview of peptidase/inhibitor units identified using peptidase database MEROPS.

Table S12. Multiheme cytochromes results checked for signal peptides using SignalP 6.0.

Table S13. Sequence dissimilarity for complete 16S rRNA genes in *VGIX01* and *JABDJQ01*, *Eisenbacteria*, *Latescibacterota*.
